# A Low-Loss 1.2 kV SiC MOSFET with Improved UIS Performance

**DOI:** 10.3390/mi14051061

**Published:** 2023-05-17

**Authors:** Lijuan Wu, Mengyuan Zhang, Jiahui Liang, Mengjiao Liu, Tengfei Zhang, Gang Yang

**Affiliations:** School of Physics & Electronic Science, Changsha University of Science & Technology, Changsha 410114, China; 15096069633@163.com (M.Z.);

**Keywords:** bipolar degradation, silicon carbide (SiC) trench MOSFET, integrated low-barrier diode, unclamped inductive switching, switching loss

## Abstract

In this article, a 1.2-kV-rated double-trench 4H-SiC MOSFET with an integrated low-barrier diode (DT-LBDMOS) is proposed which eliminates the bipolar degradation of the body diode and reduces switching loss while increasing avalanche stability. A numerical simulation verifies that a lower barrier for electrons appears because of the LBD; thus, a path that makes it easier for electrons to transfer from the N+ source to the drift region is provided, finally eliminating the bipolar degradation of the body diode. At the same time, the LBD integrated in the P-well region weakens the scattering effect of interface states on electrons. Compared with the gate p-shield trench 4H-SiC MOSFET (GPMOS), the reverse on-voltage (*V*_F_) is reduced from 2.46 V to 1.54 V; the reverse recovery charge (*Q*_rr_) and the gate-to-drain capacitance (*C*_gd_) are 28% and 76% lower than those of the GPMOS, respectively. The turn-on and turn-off losses of the DT-LBDMOS are reduced by 52% and 35%. The specific on-resistance (*R*_ON,sp_) of the DT-LBDMOS is reduced by 34% due to the weaker scattering effect of interface states on electrons. The HF-FOM (HF-FOM = *R*_ON,sp_ × *C*_gd_) and the P-FOM (P-FOM = *BV*^2^/*R*_ON,sp_) of the DT-LBDMOS are both improved. Using the unclamped inductive switching (UIS) test, we evaluate the avalanche energy of devices and the avalanche stability. The improved performances suggest that DT-LBDMOS can be harnessed in practical applications.

## 1. Introduction

Silicon carbide (SiC) has great advantages, such as high voltage and high efficiency, and potential in applications due to its superior performance compared to Si [1]. Silicon carbide (SiC) power MOSFETs have been extensively researched in recent years due to improvements in electrical properties and reliability [2]. SiC power MOSFETs are increasingly used in power electronics applications such as electric cars, photovoltaic inverters, uninterruptible power supplies, and energy distribution networks [3,4,5,6].

However, SiC MOSFETs still suffer from high loss in the body diode and the bipolar degradation issue. It has been found that the operation of the built-in body diode of SiC MOSFETs leads to performance degradation due to the production of stacking faults (SFs) originating from basal plane dislocation (BPD) in the epi-layers [7,8]. A great deal of teams put forward and authenticated monolithically integrated MOSFET and JBS/SBD diode structures [9,10,11,12,13,14] to solve it. Unfortunately, this may increase the module’s volume and parasitic capacitance [15]. Moreover, Schottky contact has poor stability at high temperatures [9]. Some structures adjust the conduction voltage drop of the body diode by varying the thickness of oxides [16,17], but a thin oxide leads to reliability issues that cannot be ignored. Some devices that integrate a low-barrier diode [18,19] have also been proposed in recent years, but they have not improved the avalanche energy of devices and the avalanche stability or conduction characteristics. The integrated channel diode is applied to planar gate MOSFET [20,21], but it is detrimental to the compact cell design and it possess strong JFET effects.

A 1.2-kV-rated double-trench 4H-SiC MOSFET with an integrated low-barrier diode (DT-LBDMOS) is proposed, which can eliminate the bipolar degradation caused by the conduction of the body diode. The integrated low-barrier diode (LBD) turns on at a low source–drain voltage, inactivating the body diode. Its turn-on loss is reduced because of the lower *Q*_rr_ and the source trench changes the capacitance distribution of the GPMOS, resulting in lower turn-off loss. Additionally, along with the introduction of the LBD, the specific on-resistance (*R*_ON,sp_) is reduced by 34% due to the weaker scattering effect of interface states on electrons. Finally, the HF-FOM (HF-FOM = *R*_ON,sp_ × *C*_gd_) and the P-FOM (P-FOM = *BV*^2^/*R*_ON,sp_) of the DT-LBDMOS are both improved. The avalanche energy and stability of the DT-LBDMOS are also improved.

## 2. Device Structure and Mechanism

**Figure 1:**
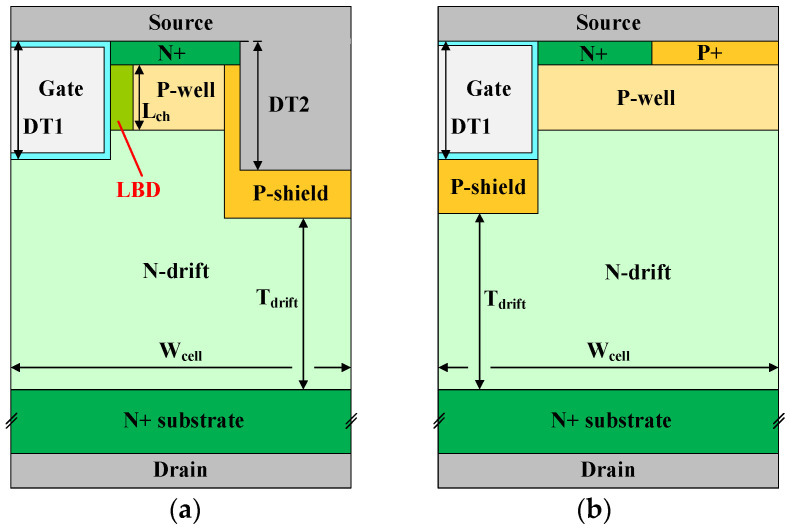
Schematic cross-sectional view of (**a**) DT-LBDMOS and (**b**) GPMOS.

**Figure 2:**
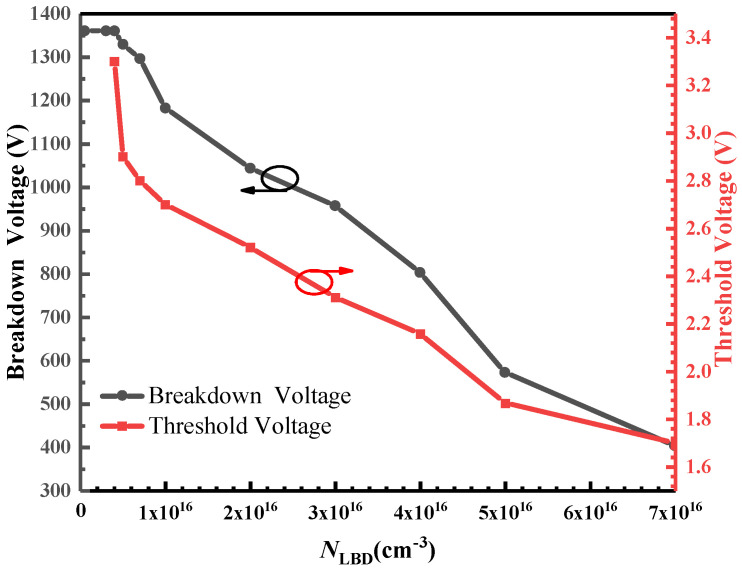
Breakdown voltage and threshold voltage under different concentrations of LBD.

The structures of the DT-LBDMOS and GPMOS are shown in Figure 1a,b, respectively. The low-barrier diode (LBD) is integrated between the trench gate and the P-well, connecting the N+ region and the drift region. The key parameters of the two devices in the simulation are listed in Table 1. The width and the length of the LBD are 0.1 μm and 0.5 μm, respectively. The thickness of the oxide layer is 50 nm and the thickness of the epitaxial layer is 12 μm. Because the PN junction between the drift region and P-shield region will undertake the breakdown voltage, the length of the drift region is 9.6 μm. The concentration of the drift region is 7 × 10^15^ cm^−3^. The depths of the gate trench and the source trench are 1.6 μm and 2 μm, respectively. The source trench is slightly deeper than the gate trench to achieve lower gate-to-drain capacitance (*C*_gd_). If the concentration of the LBD is too high, the proposed structure will conduct directly when operating in the forward blocking mode so that it cannot undertake the breakdown voltage, and considering that the threshold voltage should be kept at a safe value, which is greater than or equal to 2.5 V, the concentration of the LBD is 5 × 10^15^ cm^−3^. Through simulation, we obtained the influence of different N_LBD_ on the breakdown voltage and threshold voltage of the DT-LBDMOS as shown in Figure 2. It shows that the threshold voltage gradually decreases with the increase in N_LBD_, and the breakdown voltage remains stable at a low N_LBD_ but rapidly decreases at a high N_LBD_. Finally, when the concentration of the LBD is 5 × 10^15^ cm^−3^, the breakdown voltage of the DT-LBDMOS can reach about 1400 V and the threshold voltage of the DT-LBDMOS is about 3.3 V.

We made use of TCAD simulations to simulate the device’s electrical properties of the proposed and conventional structures. We considered some of the key models during the simulation, such as mobility saturation in the large electric field, SRH and Auger recombination, Okuto–Crowell impact ionization, bandgap narrowing, incomplete ionization, IALMob mobility degradation [22], and some temperature-dependent models and thermal boundary conditions were considered, including the thermodynamic model and the AnalyticTEP thermoelectric power model [23]. In order to make the simulation results more realistic, the traps and fixed charges at SiC/SiO_2_ interface were also considered [23].

**Figure 3:**
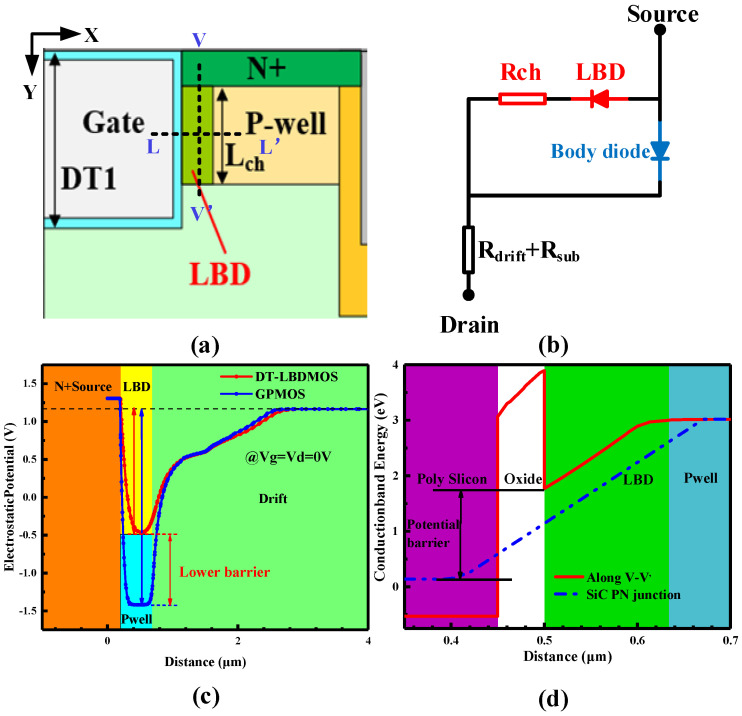
(**a**) Schematic cross-sectional view of DT-LBDMOS, (**b**) equivalent circuit diagram of third quadrant, (**c**) electrostatic potential along the V−V’ near oxide gate interface, (**d**) conduction band energy distribution along the L−L’.

The integrated low-barrier diode is the key of the proposed device. Figure 3b shows the equivalent circuit of the third quadrant of the DT-LBDMOS. The LBD is connected in parallel with the body diode, with a lower conduction voltage, so that the LBD will conduct preferentially instead of the body diode. In Figure 3c, the LBD provides a low-barrier path for electrons; the barrier height of the LBD is much lower than the barrier height of the body diode. Because the LBD connects the N+ source and drift region, we should ensure that the LBD is completely depleted in the forward blocking mode. As shown in Figure 3d, in the fully depleted LBD region, the large voltage drop caused by the high electric field leads to a downward trend in the conduction band energy from the p-well region to the oxide interface and the conduction band energy reaches the minimum at the oxide interface. Because of the lower reverse on-voltage (*V*_F_), the DT-LBDMOS possesses lower reverse conduction loss. Additionally, because the LBD operates in the unipolar mode, the bipolar degradation caused by the body diode is eliminated.

**Figure 4:**
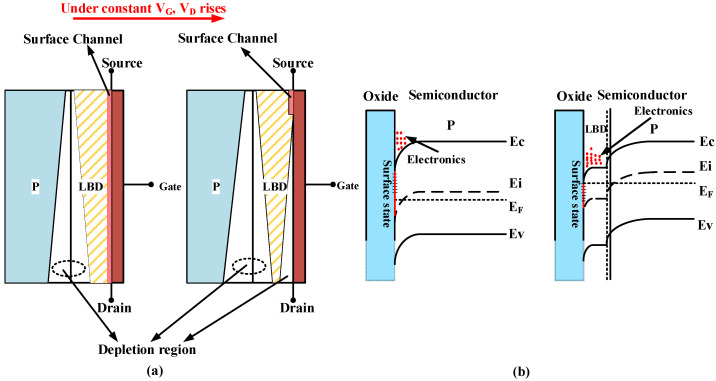
(**a**) Along with increased *V*_D_, changes in working state of DT-LBDMOS, (**b**) distributions of bandgap and electronics in on−state of DT−LBDMOS and GPMOS.

When the DT-LBDMOS operates in the on-state, the LBD can weaken the scattering effect of the SiC-SiO_2_ interface states on electrons. As shown in Figure 4a, when the DT-LBDMOS operates at a constant gate voltage *V*_G_, with the increase in drain–source voltage *V*_DS_, the surface channel is gradually depleted, DT-LBDMOS will gradually change from the surface channel conduction mode to the internal conduction mode, and the scattering effect of the SiC-SiO_2_ interface states on electrons will be weakened. When the drain voltage *V*_D_ increases to the point where the surface channel is completely depleted, the DT-LBDMOS does not enter the saturation region like the traditional device, but switches to internal conduction completely; the scattering effect of the interface states on electrons is further reduced. Figure 4b shows the energy gap and the distribution of electrons and surface states in the on-state; the electrons are distributed not only in the interface of SiC-SiO_2_ with a severe scattering effect, but also in the LBD region which is in a flat-band state. Thus, the specific on-resistance (*R*_ON,sp_) decreases.

## 3. Simulation Results and Analysis

**Figure 5:**
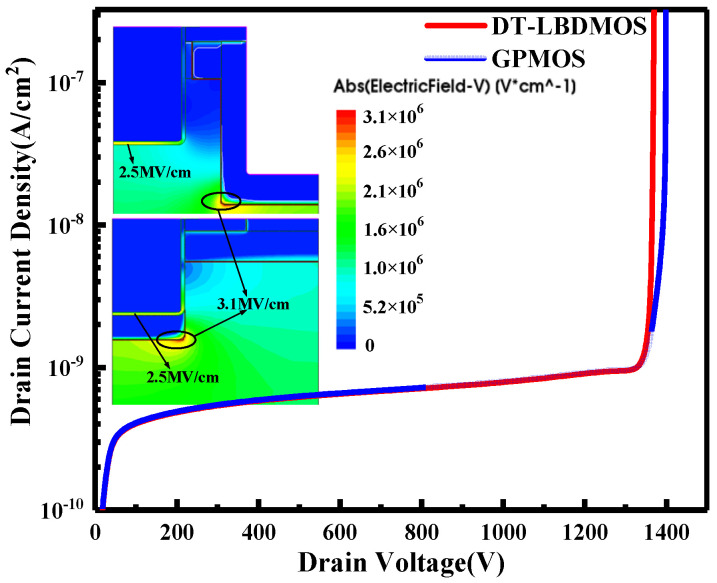
Breakdown voltage and electric field distributions of DT-LBDMOS and GPMOS.

Figure 5 shows the breakdown characteristics of the DT-LBDMOS and GPMOS. The simulation results show that the two devices are consistent in terms of breakdown voltage and electric field protection; they both achieve a 1200 V level. The peak value of the electric field appears in the PN junction formed by the P-shield region and the drift region, and as a result of Gauss’s law, the electric field inside the oxide will reach the maximum value at the bottom of the oxide. The peak values of the electric field in the oxide are both around 2.5 MV/cm, which are lower than the maximum safe electric field of 3 MV/cm. From the results, we can conclude that the oxide of both devices is protected; they both meet the design requirements for devices in the 1200 V-breakdown-voltage class.

**Figure 6:**
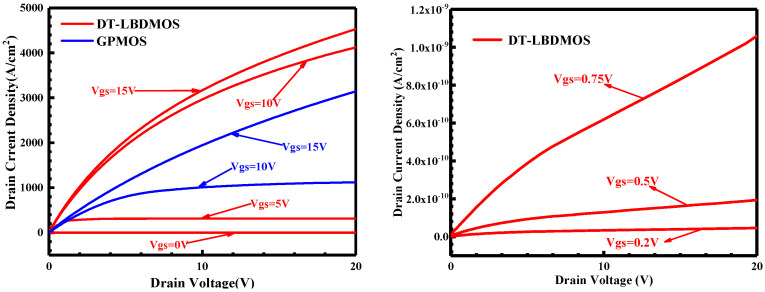
On-state output characteristics at different gate voltages of DT−LBDMOS and GPMOS.

Figure 6 shows the on-state output characteristics at the different gate voltages of the DT-LBDMOS and GPMOS. It is clear from the curves that the DT-LBDMOS possesses a higher output current density and a larger variable resistance region than the GPMOS. As mentioned above, this is a result of the weaker scattering effect of the SiC-SiO_2_ interface states on electrons in the DT-LBDMOS, and its internal conduction makes the DT-LBDMOS enter the saturation region more slowly. We take the current when the drain–source voltage reaches 1 V to calculate the specific on-resistance. The specific on-resistance (*R*_ON,sp_) of the DT-LBDMOS is 1.9 mΩ·cm^2^, which is more than 30% lower than the 2.9 mΩ·cm^2^ of the GPMOS. Finally, the P-FOM (P-FOM = *BV*^2^/*R*_ON,sp_) of the DT-LBDMOS and GPMOS is 988 MW/cm^2^ and 678 MW/cm^2^, respectively. The DT-LBDMOS has a 46% increase in P-FOM compared to the GPMOS.

**Figure 7:**
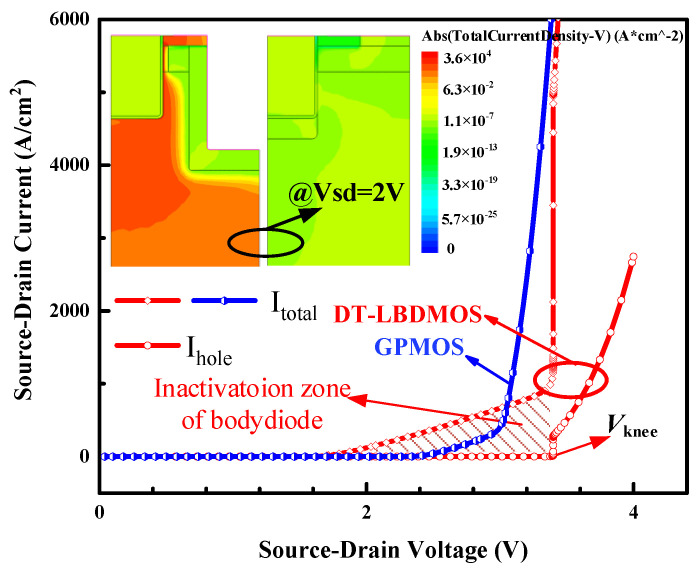
The third−quadrant output characteristics of two devices and current density distribution at *V*_sd_ = 2 V.

To test the third-quadrant characteristics of the device, a negative voltage was applied to drain electrode. Figure 7 reveals the test results; the GPMOS uses a body diode as a freewheeling diode, so that the total source–drain current includes a hole current. The DT-LBDMOS uses an LBD as a freewheeling diode; the hole current of it will not be generated at the same time as the total source–drain current. From the simulation results, the reverse on-voltage (*V*_F_) of the DT-LBDMOS and GPMOS is 1.54 V and 2.46 V, respectively. Due to the GPMOS using the body diode as a freewheeling diode, we can consider the *V*_F_ of the GPMOS the on-voltage of its body diode. The simulation results of the body diode are consistent with the typical built-in potential of a SiC p-n diode (≈−2.6 V) [24]. As shown in Figure 7, the *V*_knee_ of the DT-LBDMOS is 3.4 V, which is significantly higher than the on-voltage of the body diode. Before the source–drain voltage (*V*_SD_) reaches *V*_knee_, the DT-LBDMOS works in the unipolar mode; there is no hole current. Once the *V*_SD_ amounts to *V*_knee_, an instantaneous surge of the hole current occurs and the total current of the DT-LBDMOS rapidly reaches the total current of the GPMOS. Thus, we can conclude that the on-voltage of the body diode in the DT-LBDMOS is *V*_knee_ = 3.4 V, which is higher than the GPMOS. This proves that the LBD can inhibit the body diode, and we consider the shaded part in Figure 7 to be the inactivation zone of the body diode. In this voltage range, the body diode will not work. Finally, the DT-LBDMOS can avoid bipolar degradation and maintain the performance of the devices.

**Figure 8:**
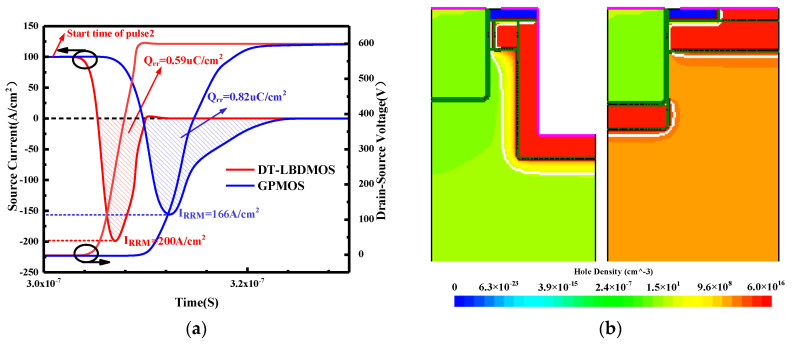

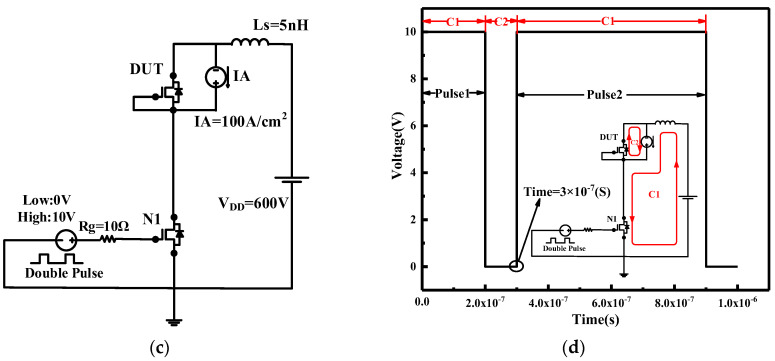
(**a**) Reverse recovery characteristics simulation results of two devices, (**b**) Hole density distributions during reverse freewheeling. (**c**) Double−pulse test circuit. (**d**) Gate pulse and current loop during test.

We chose the circuit of Figure 8c to simulate the reverse recovery characteristics of the DT-LBDMOS and GPMOS. The gate resistance *R*_g_ is 10 Ω; the switching speed of N_1_ can be changed by changing the resistance value of *R*_g_, thus changing the reverse recovery speed of devices being tested [25,26]. Figure 8d shows the pulse information applied to the gate during the test and the handover process of the circuit’s current loop. Pulse1 starts at time = 0 (s) and ends at time = 2 × 10^−7^ (s); pulse2 starts at time = 3 × 10^−7^ (s) and ends at time = 9 × 10^−7^ (s). Pulse1 makes N1 conductive and the current loop is C1; after pulse1 N1 switches off, the circuit performs reverse freewheeling through the DUT, and the current loop is C2. Then, pulse2 causes N1 to conduct again, the current loop turns to C1, and the DUT switches from the on state to the off state; at that time, the DUT generates a reverse recovery current, which is the reverse recovery characteristic of the DUT. The curves in Figure 8a show the reverse recovery characteristics of the two devices; the reverse recovery process occurs after pulse2 starts, lasting for tens of nanoseconds, so the time range of Figure 8a is 3 × 10^−7^ (s) to 3.3 × 10^−7^ (s). From the simulation results in Figure 8a, the reverse recovery time *t*_rr_ of the DT-LBDMOS is obviously shorter than that of the GPMOS. However, the peak value of the reverse recovery current (IRRM) for the DT-LBDMOS will be slightly higher than the GPMOS; the reason is the reverse recovery current of the GPMOS is because free carriers stored in the drift region during the conduction of the body diode were extracted, but the reverse current of the DT-LBDMOS is due to the formation of a charging displacement current of source–drain capacitance. The *C*_ds_ of the DT-LBDMOS is large and causes a higher reverse recovery current. We integrated the reverse current below the *x*-axis with time to obtain the reverse recovery charge (*Q*_rr_); the *Q*_rr_ of the two devices is represented by the shaded parts that are of different colors. The reverse recovery charge (*Q*_rr_) of the DT-LBDMOS is 0.59 uC/cm^2^, which is a 28% decrease compared to the 0.82 uC/cm^2^ of the GPMOS. Figure 8b shows the hole density of the DT-LBDMOS and GPMOS during the reverse conduction and reverse recovery (at *I*_SD_ = 100 A/cm^2^). As mentioned above, the performance of the devices will be degraded due to the conduction of the body diode, so we prefer the SiC MOSFET to operate in unipolar mode during reverse freewheeling. We can conclude that the DT-LBDMOS operates in the unipolar mode from Figure 8b as there are extremely few holes injecting into the drift region.

**Figure 9:**
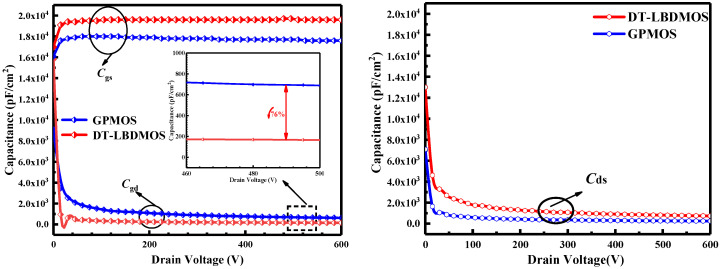
The *C*_gs_, *C*_gd_ and C_ds_ simulation results of DT-LBDMOS and GPMOS.

The capacitances of the DT-LBDMOS and GPMOS are shown in Figure 9; the *C*_gd_ of the DT-LBDMOS and GPMOS extracted at *V*_DS_ = 500 V is 180 and 735 pF/cm^2^, respectively. Additionally, the *C*_gs_ and *C*_ds_ of the DT-LBDMOS are slightly higher than that of the GPMOS. This is because the source trench is slightly deeper than the gate trench, which weakens the capacitive coupling between the gate and drain and transfers it to the source, and the part of the source extending to the drift region makes the coupling area between the source and drain increase. As a result, the HF-FOM (HF-FOM = *R*_ON,sp_ × *C*_gd_) of the DT-LBDMOS decreases from 2131.5 mΩ·pF of the GPMOS to 342 mΩ·pF, which is a decrease of 84%.

**Figure 10:**
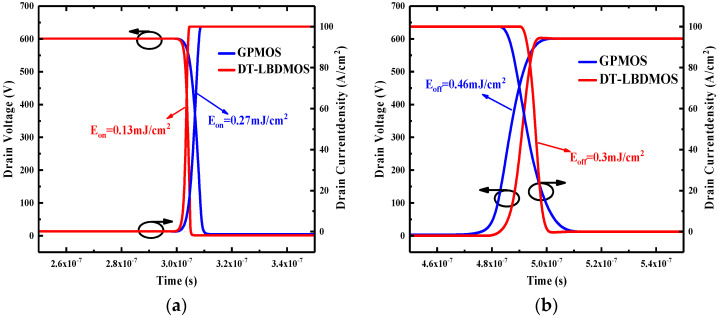
(**a**) Devices’ turn−on wave forms and loss, (**b**) Devices’ turn−off wave forms and loss.

Figure 10 compares the switching characteristics between the DT-LBDMOS and GPMOS; the turn-on loss (*E*_ON_) of the DT-LBDMOS is only 0.13 mJ/cm^2^, showing a 52% reduction compared to the GPMOS. This difference is due to the reduction in the reverse recovery charge, allowing the DT-LBDMOS to conduct faster, resulting in a lower turn-on loss. The turn-off loss (*E*_OFF_) of the DT-LBDMOS is 0.3 mJ/cm^2^, showing a 35% reduction compared with the GPMOS, due to the reduction in *C*_gd_ and HF-FOM of the DT-LBDMOS.

**Figure 11:**
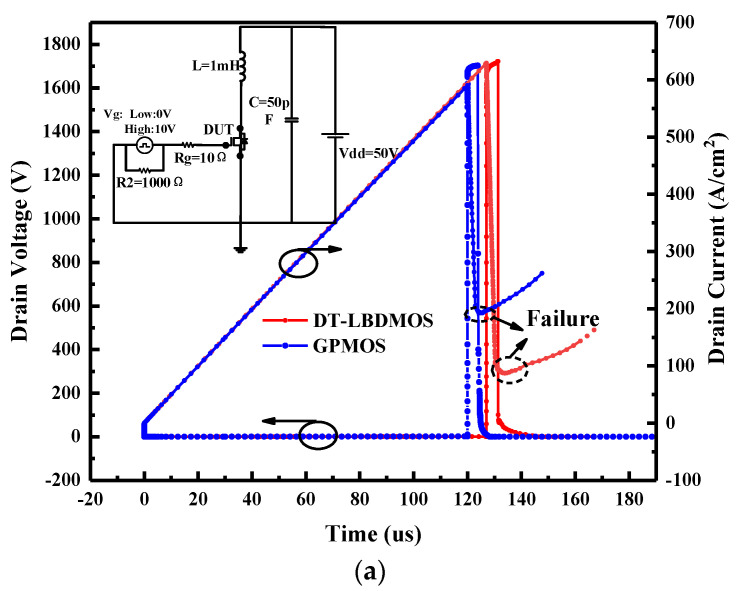

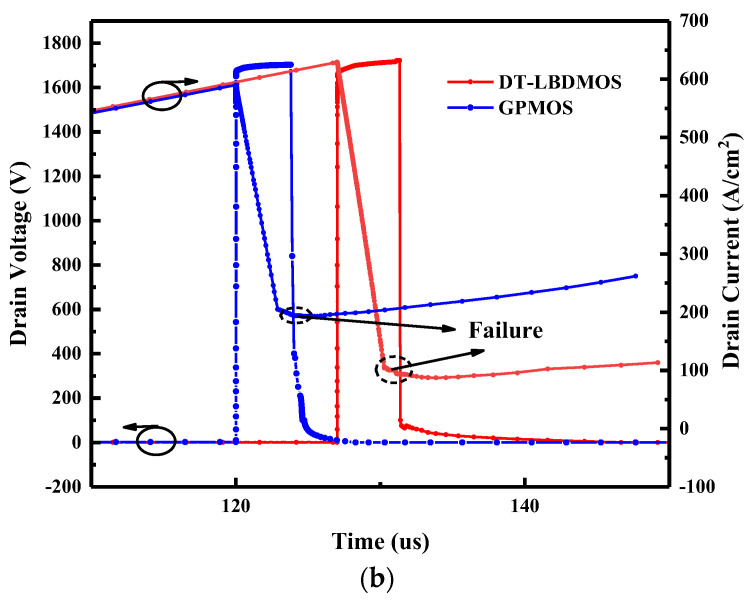
(**a**) The test circuit and UIS simulation results at failure of DT−LBDMOS and GPMOS. (**b**) scale of around 120−150 μs.

In the application of power conversion, the energy stored by the external or parasitic inductor of the MOSFET is released when the devices are turned off, forcing the devices to avalanche. The high voltage and large current will cause impacts which can easily lead to device failure. Therefore, we evaluated the avalanche energy and the avalanche stability of the DT-LBDMOS and GPMOS under unclamped inductive switching (UIS) to ensure that the devices can work regularly under the extreme environment of the system. Figure 11 shows the output waveforms of the two devices at critical failure and the UIS test circuit. The values of *V*_DD_ and *L* are 50 V and 1 mH [27], respectively. It shows that the avalanche time of the DT-LBDMOS is a little longer than that of the GPMOS, but in fact, the conditions of the simulation are the same. Further analysis is needed to explain this result.

**Figure 12:**
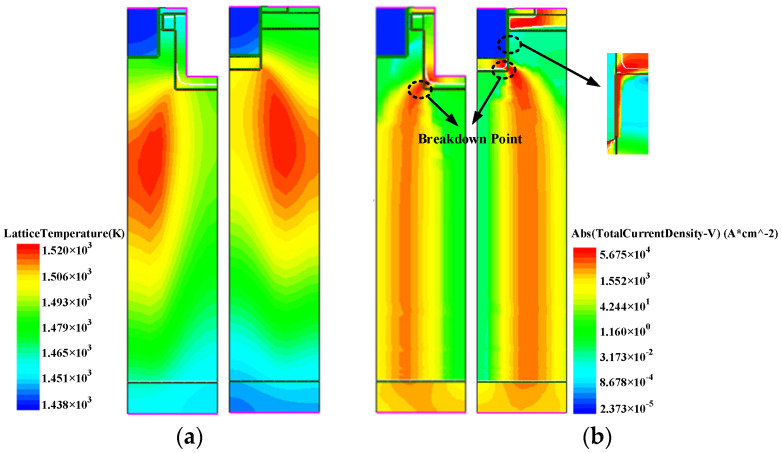
The temperature and current distributions at failure of DT−LBDMOS and GPMOS. (**a**) Temperature distributions. (**b**) Current distributions.

Figure 12 shows the temperature and current distributions of the two devices at critical failure. Figure 12a shows the temperature distributions of the DT-LBDMOS and GPMOS; the heat generation centers are located in the central region of the drift under the source and gate, respectively. Figure 12b shows the current distributions of the DT-LBDMOS and GPMOS; the current does not flow through the N+ source region and the simulated drain voltage does not reveal a sudden drop in voltage due to secondary breakdown. From Figure 12, we can conclude that the failure mechanisms of the two devices are thermal failure but not triode failure. In high-temperature and high-electric-field environments, traps at the interface of SiC-SiO_2_ are very likely to capture low-energy carriers, while high-energy carriers can enter into SiO_2_ to form more interface traps, known as hot carrier injection effects. From Figure 12a,b, the current at failure of the GPMOS flows through the whole SiC-SiO_2_ interface and the temperature near the gate of the GPMOS is higher than that of the DT-LBDMOS; the differences are caused by the different breakdown point. We infer that the different test results of the UIS test were caused by the different temperature distribution and current distribution of the two devices. This led to heavier hot carrier injection in the SiC-SiO_2_ interface of the GPMOS.

**Figure 13:**
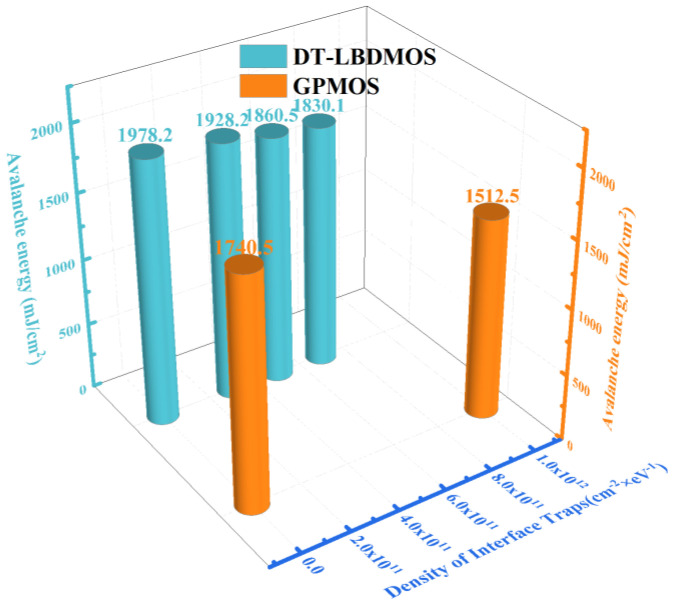
Simulation results of avalanche energy with different interface trap densities of of two devices.

To verify whether the interface states caused by the hot carrier injection effect will affect the UIS test results of the two devices, we added traps in the simulations to imitate the results of the hot carrier injection effect [28]. Figure 13 reveals the simulation results of avalanche energy with different density in the interface traps. The avalanche energy of device was calculated from the energy stored in the inductor and the formula is *E*_AS_ = 1/2 × *L* × *I*_peak_^2^. As the density of the interface traps increases, the avalanche energy of the DT-LBDMOS gradually decreases. Additionally, because of the weaker hot carrier injection effect, we added fewer interface trap densities to the DT-LBDMOS. The simulation results confirm that the interface states caused by the hot carrier injection effect will reduce the avalanche energy. The simulation result without interface traps was calculated from the simulation in Figure 11. Thus, the DT-LBDMOS has better avalanche energy and stability than the GPMOS. For the DT-LBDMOS, a weaker hot carrier injection effect in practical application will lead into fewer surface states in the interface of SiC-SiO_2_, and in a repeated UIS test, fewer surface states in the interface of SiC-SiO_2_ will also reduce the electric field in the oxide. Thus, it can be concluded that the DT-LBDMOS possesses higher UIS stability and oxide reliability under high voltage and current.

We compared the HF-FOM (HF-FOM = *R*_ON,sp_ × *C*_gd_) and the P-FOM (P-FOM = *BV*^2^/*R*_ON,sp_) between the DT-LBDMOS and several real devices. Table 2 shows the results. The real devices were fabricated in [29]; they can represent a high level of currently commercially available devices. According to Table 2, we can conclude that the DT-LBDMOS has sufficient application potential.

**Figure 14:**
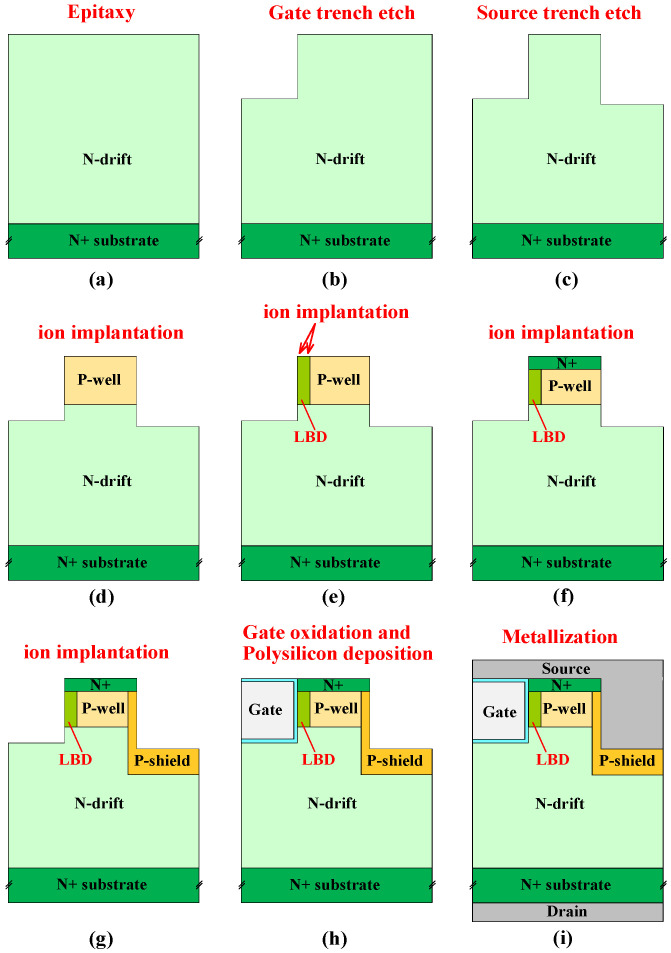
Fabrication process flow of DT−LBDMOS. (**a**) Epitaxial growth. (**b**) Gate trench etching. (**c**) Source trench etching. (**d**) P-well region implantation. (**e**) LBD region implantation. (**f**) N+ region implantation. (**g**) P-shield region implantation. (**h**) Gate oxidation and polysilicon deposition. (**i**) Metallization.

The core process flow of SiC device fabrication lines is deposition with a mask, photoetching, etching with a mask, ion implantation, high-temperature activation, isolated oxidation, gate oxidation, polysilicon gate deposition, and metallization. Based on this, we propose a fabrication process for the DT-LBDMOS. The specific process is growing the N-type SiC drift region on the N+ substrate (Figure 14a), etching gate trench (Figure 14b), next etching source trench (Figure 14c), forming a P-well region through ion implantation (Figure 14d), after that forming an LBD region through ion implantation as well (Figure 14e), forming an N+ region through ion implantation (Figure 14f), and forming a P-shield region through ion implantation (Figure 14g). Next, thermal oxidation is performed to grow the oxide layer and fill poly silicon (Figure 14h), and the drain and source electrodes are metallized (Figure 14i).

## 4. Conclusions

With higher performance requirements for SiC MOSFETS, a double-trench 4H-SiC MOSFET with an integrated low-barrier diode (DT-LBDMOS) is proposed and simulated by TCAD. The simulation results reveal that the DT-LBDMOS eliminates the bipolar degradation of the body diode, reducing the reverse on voltage (VF) by 41.6% from 2.46 V to 1.54 V, and the reverse recovery charge (Qrr) is reduced by 28% from 0.82 uC/cm^2^ to 0.59 uC/cm^2^. Meanwhile, the switching characteristics, P-FOM, and HF-FOM are improved. The avalanche energy and stability of the DT-LBDMOS are better than the GPMOS, and under a high voltage and current, the DT-LBDMOS possesses higher gate oxide reliability.

## Figures and Tables

**Table 1:** Key parameters of the device in simulation.

Parameters	Descriptions	Values
***W_LBD_***	LBD width (μm)	0.1
***t*_ox_**	Gate oxide thickness (nm)	50
***W_cell_***	Half-cell width (μm)	2.85
***N_drift_***	Drift region concentration (cm^−3^)	7 × 10^15^
***L_ch_***	Channel length (μm)	0.5
***N_LBD_***	LBD concentration (cm^−3^)	5 × 10^15^
***T_drift_***	Thickness of drift region (μm)	9.6
***DT*_2_**	Depth of source trench (μm)	2.0
***N_SUB_***	Substrate region concentration (cm^−3^)	1 × 10^19^
***N_P-shield_***	P-shield region concentration (cm^−3^)	1 × 10^18^
***N_POLY_***	Poly region concentration (cm^−3^)	1 × 10^21^
***N_P-well_***	P-well region concentration (cm^−3^)	3.5 × 10^17^
***N_P+_***	P+ region concentration (cm^−3^)	1 × 10^19^
***N_N+_***	N+ region concentration (cm^−3^)	1 × 10^19^

**Table 2:** Comparison between DT-LBDMOS and several real devices.

Device	*BV* (V)	*R*_ON,sp_ (mΩ·cm^2^)	*C*_gd_ (pF/cm^2^)	P-FOM (MW/cm^2^)	HF-FOM (pF/cm^2^)
Lin_J0.7 Acc [29]	1628	5.61	106	472	595
Sqr_J1.1 Acc [29]	1338	5.53	392	323	2168
Hex_J1.1 Acc [29]	1436	5.50	386	374	2123
Hex_J0.7 Acc [29]	1620	4.87	286	538	1393
O_J1.1 Acc [29]	1605	12.82	35	200	449
O_J1.1_C Acc [29]	1605	8.47	48	304	407
DT-LBDMOS	1420	1.9	180	988	342

## Data Availability

Not applicable.
